# Media acidification by *Escherichia coli *in the presence of cranberry juice

**DOI:** 10.1186/1756-0500-2-226

**Published:** 2009-11-12

**Authors:** Brandy J Johnson, Baochuan Lin, Robert A Rubin, Anthony P Malanoski

**Affiliations:** 1 Center for Bio/Molecular Science & Engineering, Code 6900, Naval Research Laboratory, Washington, DC 20375, USA; 2 Independent Researcher, 8620 Portafino Place, Whittier, CA 90603, USA

## Abstract

**Background:**

The inhibition of *Escherichia coli *growth in the presence of *Vaccinium macrocarpon *has been extensively described; however, the mechanisms of this activity are not well characterized.

**Findings:**

Here, *E. coli *was grown in media spiked with cranberry juice. The growth rate and media pH were monitored over more than 300 generations. The pH of the growth media was found to decrease during cell growth. This result was unique to media spiked with cranberry juice and was not reproduced through the addition of sugars, proanthocyanidins, or metal chelators to growth media.

**Conclusion:**

This study demonstrated that factors other than sugars or proanthocyanidins in cranberry juice result in acidification of the growth media. Further studies are necessary for a complete understanding of the antimicrobial activity of cranberry products.

## Findings

Early studies focusing on the antimicrobial activity of *Vaccinium macrocarpon *(cranberry) implicated the acidifying effect the juice had on urine and/or increased excretion of the cranberry metabolite hippuric acid as potential antimicrobial mechanisms [1-3]. Studies demonstrated that consumption of cranberry juice did lower urinary pH, however, no direct bacteriostatic effect was observed from the urine samples [2,3]. Other studies have reported no change in urinary pH upon consumption of cranberry juice [4,5]. More recently, the evidence for the role of urinary acidification by cranberry juice has been called into question [5-7]. Rhee suggests that ascorbic acid in the cranberry juice may facilitate non-enzymatic generation of nitric oxide (NO) which possesses potent antimicrobial activities [7]. The growth of *E. coli *was markedly inhibited in mildly acidified urine when nitrite was present [8]. Current studies tend to implicate the anti-adhesive properties of the A-type proanthocyanidins (PACs) found in cranberries in the reduction of urinary tract infection as well as in health benefits related to the prevention of oral biofilms and stomach ulcers [6,9-12]. In addition to these mechanisms of action, alterations to the growth rate of bacteria were implicated in contributing to the overall antimicrobial activity observed [13]. Many recent studies have focused on determining the effects of a single compound or reported mechanism of action, most commonly the anti-adhesive action. While this approach is often the only feasible path, it can lead to a lack of understanding regarding the relative roles of the mechanisms. In addition, studies which evaluate only a single compound or mechanism may overlook the simultaneous or synergistic action of multiple compounds or mechanisms.

We have recently described the impact of cranberry juice and PACs on the morphology of *E. coli *when present as part of the growth media [14]. Changes resulting from physical interactions between bacterial cells and components of cranberry juice as well as from changes in gene expression were observed. Another study demonstrated the decrease in *E. coli *growth rate over successive batch cultures in cranberry juice-spiked media. It was noted that a number of changes in the gene expression profile of *E. coli *appeared to be related to the metal chelation capacity of the proanthocyanidin components of cranberry juice. (Lin, et al.: Iron chelation by cranberry juice and its impact on *Escherichia coli *growth, submitted) A noted change was the expression of a gene responsible for adaptive acid tolerance response (asr c1989) in *E. coli*. The expression of acid shock protein has previously been reported to increase gradually with a decrease in pH [15]. This result indicated that *E. coli *was modulating gene expression in response to acid stress when exposed to cranberry juice *in vitro*. The observed increase in expression of acid shock protein was interesting since all growth media was verified to have an initial pH of 7.4.

**Figure 1:**
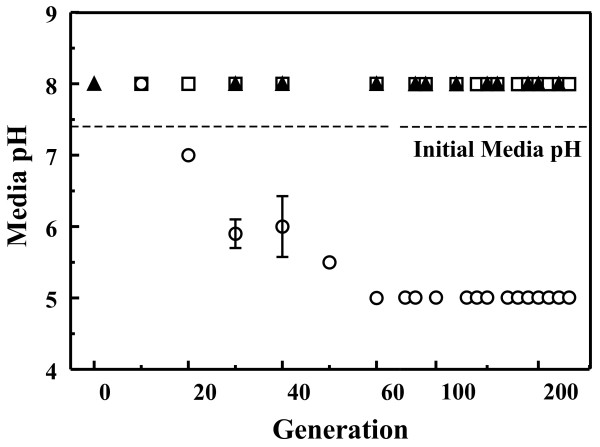
**Media pH**. The pH of all media was initially 7.4. The final pH of cultures in cranberry juice spiked media (10%, circle) decreases over the first 6 batch cultures (~60 generations) levelling off to a value of 5.0. No decrease in pH occurs for cells grown in LB broth (triangle) or PAC-spiked LB broth (0.05 mg/mL, square). Where not visible, error bars are contained within the point.

In an attempt to understand the up regulation of genes associated with acid shock observed in previous studies, the media pH for *E. coli *cultured in LB broth with 10% cranberry juice was monitored over 300 generations of bacterial growth. The juice used to spike the LB broth was first neutralized to pH 7.4 through the addition of sodium hydroxide. Neutralization of the media was necessary as the bacteria would not grow in the acidic juice. Media pH was verified following addition of the juice. The resulting growth media was identical to that used during the originally reported experiments [14]. (Lin, et al. submitted manuscript) The concentration of cranberry juice was fixed at 10% due to the lack of bacterial growth at higher cranberry juice concentrations (> 25%). The cranberry juice used here and in previous experiments was filter-sterilized Mountain Sun 100% Cranberry Juice purchased at single strength concentration. Cultures consisted of 12 mL of growth media in 50 mL polypropylene tubes which were inoculated using a 1 μL loop (approximately 10 generations per tube). Media pH was monitored using Baxter pHIX pH test strips. The pH of the media was found to decrease to pH 5.0 after generation 60 (Figure 1). The change in pH at generation 60 correlated with the observed up regulation in the acid shock protein. (Lin, et al. submitted manuscript) Subsequent batches showed a decrease in media pH beginning during mid-log phase growth. These changes in media pH were not observed for *E. coli *grown in LB broth (Figure 1).

In order to elucidate if media acidification resulted from the presence of PACs, media was spiked with 10% of a 50 μg/mL PAC solution. PACs were purified from fresh cranberries as described previously [16]. Growth media with a PAC content similar to that of cranberry juice spiked media was prepared using 10% of a 50 μg/mL PAC solution. PAC concentrations were verified using a radial diffusion assay [17]. The pH of this media over all generations was identical to that of bacteria cultured in LB broth (Figure 1) though the doubling time of the cultures was significantly lengthened. (Lin, et al. submitted manuscript) Because PACs are polymers composed of monomeric flavanoid subunits such as catechin and epicatechin, catechin was used as a growth media modifier (1.4 mM). Again, the growth rate of the *E. coli *was observed to slow, but the media pH conformed to the behavior observed for standard LB broth. These results implicated mechanisms unrelated to the PAC content of cranberries in media acidification. In order to further verify that metal chelation was not a causative factor in the changing media pH, ethylenediaminetetraacetic acid (EDTA) was added to LB broth (1 mM). While the presence of EDTA was found to increase the doubling time of *E. coli*, it did not produce changes in media pH. These results suggested that other compounds, such as sugar content, were involved in the reported activities of cranberry and cranberry products.

Based on averages collected by a number of different studies, 5.4 g sugar per 100 g juice is a reasonable estimate of the sugar content of single strength cranberry juice with an approximate ratio of 3.9 for glucose to fructose [18]. Growth media was modified through the addition of glucose (24 mM) and fructose (6 mM) to provide an approximate equivalent for the sugar content of the juice-spiked media. As expected, the growth rate of *E. coli *in this media was more rapid than growth in standard LB broth, but the pH of the media followed the profile observed when cells were grown in standard media. These results indicated that the additional sugar content of the media did not result in the activation of fermentation pathways in the bacteria.

Although the mechanisms for inhibiting bacterial growth could be attributed in large part to iron depletion from PAC components and, to a lesser degree, to direct disruption of metabolic enzymes, it is not clear whether together they account for all the growth inhibition noted in the experiments. (Lin, et al. submitted manuscript) Here, we found that culturing bacteria in 10% cranberry juice results in acidification of the media, an effect that is not observed when the bacteria is cultured in media spiked with many of the well characterized components of cranberry juice. It is not clear if this phenomenon is related to the urine acidification noted in some studies upon ingestion of cranberry juice. It is important to note that, like many *in vitro *studies directed at understanding the interactions between *E. coli *and cranberry juice/components, these studies do not reflect conditions relevant to *in vivo *activities. The mechanisms at work in this type of *in vitro *study, therefore, may not correlate to the action of cranberry juice and its constituents observed *in vivo*. The causative factors in the acidification of the growth media have not been identified, though several possible mechanisms have been eliminated. This study illustrates the complexity of the interactions between bacterial cells and the mixture of compounds found in cranberry juice. Further studies directed toward understanding the antimicrobial action of cranberry juice are necessary to elucidate the relative roles of the several mechanisms known to be in operation.

## Competing interests

There are three pending patent applications peripherally related to the work presented here on which BJJ and BL are named as inventors. These patents are all related to the interaction of PACs from cranberry with the lipopolysaccharide component of bacterial cells.

## Authors' contributions

BJJ conceived of the study, was responsible for bacterial culture and sample preparation, and drafted the manuscript. BL carried out the molecular genetic studies and participated in conception of the study and drafting the manuscript. RAR and APM participated in analysis of gene expression results. All authors read and approved the final manuscript.
